# Carcinomas of Distal Fallopian Tube and Their Association with Tubal Intraepithelial Carcinoma: Do They Share a Common “Precursor” Lesion? Loss of Heterozygosity and Immunohistochemical Analysis Using PAX 2, WT-1, and P53 Markers

**DOI:** 10.5402/2011/858647

**Published:** 2010-12-15

**Authors:** Mamatha Chivukula, Leo A. Niemeier, Robert Edwards, Marina Nikiforova, Geetha Mantha, Kim McManus, Gloria Carter

**Affiliations:** ^1^Department of Pathology, Magee Womens Hospital of UPMC, 300 Halket Street, Pittsburgh, PA 15213, USA; ^2^Department of Gynecologic Oncology, Magee Womens Hospital of UPMC, 300 Halket Street, Pittsburgh, PA 15213, USA; ^3^Molecular Pathology, Magee Womens Hospital of UPMC, 300 Halket Street, Pittsburgh, PA 15213, USA

## Abstract

As the role of distal fallopian tube as organ of serous carcinogenesis is emerging, additional literature on the role of tubal intraepithelial carcinoma (TIC) as a precursor lesion in a subset of primary peritoneal serous carcinomas (PPSC is emerging as well. TIC although fallopian tube in origin can be genetically related to ovarian/peritoneal carcinomas. The role of PAX2 in primary fallopian tube carcinomas (PFTC)/PPSC is yet to be defined. The aim of our study was to understand if the biologic properties of tumors arising in the distal fallopian tube that remain as PFTC are different when they seed on to the peritoneal surface (PPSC). A panel of 6 polymorphic microsatellite markers corresponding to p53, PAX2, and WT1 tumor suppressor genes were studied. Invasive carcinomas as well as TIC arising in the distal fallopian tube when remain as PFTC appears to exhibit different LOH patterns in comparison to PPSC. PAX 2 LOH patterns might represent a “hidden PAX 2 signature” analogous to p53 signatures. PAX 2 might be an emerging marker for detection of early serous carcinomas particularly in BRCA + women.

## 1. Introduction

Ovarian cancer is divided into two (2) histopathologic groups with distinct molecular pathogenesis as well as propensity for extra ovarian spread. Group 1 includes tumors of borderline malignancy, low-grade serous carcinomas, endometrioid and mucinous carcinomas. These tumors arise from ovarian parenchyma, confined to one or more cysts, and do not typically originate from or involve the ovarian surface and often exhibit BRAF, KRAS, P-TEN, b-catenin mutations. Group 2 includes serous carcinomas that arise from the ovarian surface epithelium or mullerian inclusions, fallopian tube mucosa, and mullerian epithelium in peritoneal cavity and often exhibit p53 mutations; these tumors are rapidly evolving and lethal [1]. Primary fallopian tube carcinomas (PFTCs) that arise from the mucosa of the fallopian tube, accounts for less than 1% of all female genital tract carcinomas [2]. Morphologic features such as dominant, centrally located mass in fallopian tube, presence of in situ carcinoma, absence of coexisting endometrial carcinoma, minimal ovarian involvement, qualifies these tumors as PFTC. Histologically, these tumors are predominantly of “serous” morphology. Primary peritoneal serous carcinomas (PPSCs) though morphologically resemble ovarian serous carcinomas (OSCs); the ovaries are either normal or minimally involved [2]. Coexisting intraepithelial neoplasia is a prerequisite for PFTC diagnosis; on contrary, PPSC are often diagnosed in the absence of an “in situ” lesion. Based on the tumor distribution, these tumors are interclassified as OSC or PPSC. Tubal intraepithelial carcinoma (TIC) is neoplastic transformation of benign tubal epithelium. Presence of TIC designates tube as “primary tumor site”. Recent studies by Crum et al. have shown the association of TIC in 47% (5/8) of PPSC and 37% (19/39) OSC cases [3–5]. The group has also established TIC as an earliest manifestation of pelvic serous carcinoma in both BRCA+/sporadic tubal carcinomas [3, 6]. As the role of distal fallopian tube as an organ of serous carcinogenesis is emerging, the primary aim of our study was to understand if the biologic properties of tumors arising in distal fallopian tube that remain as PFTC are different when seeds on peritoneal surface (PPSC). TIC though fallopian tube in origin can be genetically related to ovarian/peritoneal carcinomas, and our secondary goal was to understand the biologic properties of TIC in association with PFTC from PPSC w/associated TIC.

## 2. Materials and Methods

 MWH pathology files were searched between the years January 2006— December 2009. All tumors that met WHO 2001 criteria for PPSC were selected [2].

The criteria included the following

- Both ovaries normal size/enlarged by benign process.
- Extra ovarian involvement > surface of ovaries.
- Ovarian involvement-nonexistent—confined to surface w/no stromal invasion (<5 × 5 cm).

SEE-FIM protocol, a procedure for sectioning the entire fimbriated end (SEE-FIM protocol) in prophylactic oophorectomy specimens tubes, are first fixed for 4–6 hours: the fimbriated end (a) is amputated, (b) open-sectioned longitudinally to expose the maximum surface area, (c) submitted in toto with the remainder of the tube sectioned at 2-3 mm intervals, was adopted at MWH in 2006 [7].

PPSC were grouped into *tumors with associated TIC* in the adjacent fallopian tubes and tumors *without associated TIC.*

All tumors that met the WHO criteria for PFTC were selected as well [2]. 

Hematoxylin & Eosin (H&E) slides were reviewed and the diagnosis was confirmed. 

**Figure 1:**
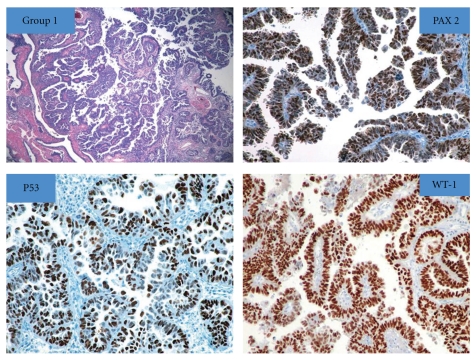
Hematoxylin and Eosin section of primary fallopian tube carcinoma (PFTC) with high grade serous papillary morphology: “nuclear” expression for PAX 2, p53, and WT-1 immunostains.

**Figure 2:**
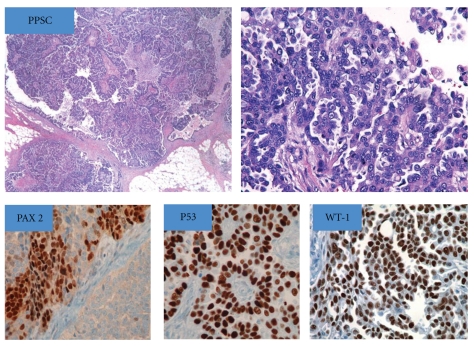
Hematoxylin and Eosin (H&E) section of primary peritoneal serous carcinomas (PPSC) with high-grade serous papillary morphology: “nuclear” expression for PAX 2, p53, and WT-1 immunostains.

**Figure 3:**
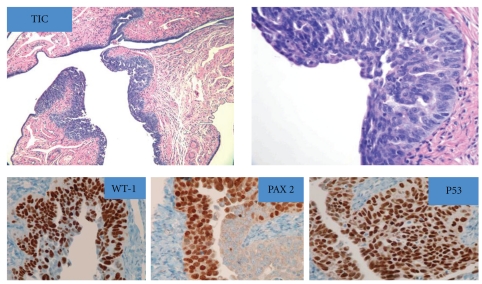
Hematoxylin and Eosin (H&E) section of tubal intraepithelial carcinoma (TIC) with stratified epithelium, marked nuclear atypia associated with a subset of PPSC as well as PFTC: “nuclear” expression for PAX 2, p53, and WT-1 immunostains.

Figures 1, 2, and 3 show the histologic and IHC expression for PAX 2, WT-1, and p53 markers.

Representative tumor blocks as well as associated TIC in the corresponding fallopian tube sections were selected. 

Wilm's tumor gene 1 (WT-1), p53, and PAX 2 gene markers were selected for loss of heterozygosity (LOH) and immunohistochemical (IHC) analysis. 

### 2.1. Loss of Heterozygosity (LOH) 

#### 2.1.1. Microdissection and DNA Isolation

One or two paraffin blocks were selected to represent tumor and normal tissue from each patient. Ten (10) serial sections were obtained from paraffin-embedded tissue blocks. The first and last sections were stained with H&E to confirm that the intervening blank slides contained the lesional material. Tissue fragments were microdissected under stereomicroscopic visualization from 6–8 blank deparaffinized slides for each of the marked targets. Areas that contained large amounts blood, necrosis, or contaminating normal tissue were avoided during microdissection. DNA was isolated using DNEasy tissue extraction kit (Qiagen, Chatsworth, Calif.; USA). PCR amplification was performed using fluorescently labeled primers flanking microsatellite repeat polymorphisms located in close proximity to the specific genes of interest. Six microsatellite loci situated adjacent to three known tumor suppressor genes on chromosome 10q (PAX2 D10S.520, D10S.1173), 11p (WT-1 D11S.2370, D11S.995), and 17p (p53 D17S.1844, D17S.516) were assessed.

### 2.2. Loss of Heterozygosity (LOH) Analysis

**Figure 4:**
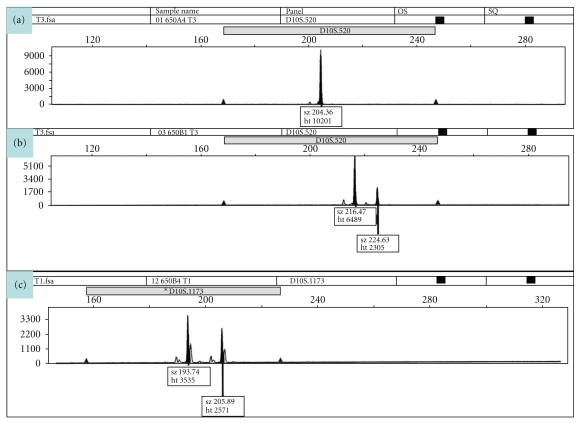
This figure shows the Loss of Heterozygosity (LOH) patterns of the normal (a) in comparison to the corresponding tumor (b), (c).

Amplification products were detected using fluorescence capillary electrophoresis and Gene Mapper Software 4.0 (ABI 3730, Applied Biosystems, Foster City, CA). Allelic peak heights of each electropherogram in relative fluorescence units were analyzed. The presence or absence of allelic imbalance was accessed by comparing allelic peak heights for each tumor sample with corresponding normal tissue. The sample was considered as noninformative (NI) if the tissue sample demonstrates only a single peak. Individuals with a heterozygous allele pattern (two peaks) were designated as informative for that particular locus (Figure 4). Loss of heterozygosity (LOH) was calculated using the following formula: LOH = allele ratio [tumor]/allele ratio [normal]). The sample was considered to have high level of allelic imbalance if the allelic ratio for a specific microsatellite marker was less than 0.5 or greater than 2.0 and to have low level of allelic imbalance when the peak height ratios fall into the range of 0.5–0,7 or 1.65–2.0.

### 2.3. Immunohistochemistry (IHC)

Immunohistochemical stains were performed on the blank sections on the corresponding paraffin-embedded block selected for LOH analysis. The details of the antibodies are specified in Table 1. A strong “nuclear” staining is considered positive for all the three antibodies WT-1, p53, and PAX 2. The intensity of staining (IS) was graded as negative (0), weak (1), moderate (2), and strong (3), and proportion of positive staining cells (PS) was recorded as 0%–5% (1), 1%–20% (2), 21-80% (3) and greater than 80% (4).

A cumulative score (CS) is calculated incorporating PS as well as IS. The score is derived by the summation of PS and IS, ranged from 0 to 7 and is further divided into *Negative* (0), *Weak* (1-2); *Moderate* (3–5); *Strong* (6–7).

## 3. Results

All the tumors are categorized into three groups.

### 3.1. Group 1: PFTC in the Background of TIC (n-10)

This group included all cases of primary fallopian tube carcinomas (PFTCs). All tumors (100%) were present in the background of TIC. 

Table 2 shows the clinical and pathologic features of the PFTC (Group 1) cases.

#### 3.1.1. IHC and LOH Results of Group 1

**PAX 2**

there is heterogeneity in PAX 2 immunostain expression in both TIC as well as the adjacent invasive carcinoma. A moderate-strong staining is seen in 40% (4/10) cases of both TIC as well as the adjacent invasive carcinoma. LOH was seen in up to 30% (3/10) in the invasive carcinomas. The foci of TIC were able to be captured for LOH analysis in four (4) out of ten (10) cases. LOH was in 75% (3/4) cases.

**WT-1 and p53**

immunohistochemical expression for WT-1 and p53 were similarly seen consistently. A strong nuclear staining is seen in 100% (10/10) of all TIC and 80% (8/10) adjacent invasive carcinoma. LOH was seen in up to 50% (2/4) of TIC and 30% (3/10) of invasive carcinomas.

### 3.2. Group 2: PPSC without Associated TIC (PPSC/-) (n-9)

This group includes all cases of PPSC without associated TIC in the fallopian tubes. 

#### 3.2.1. IHC and LOH Results of Group 2

**PAX 2**

there is heterogeneity in the expression of PAX 2 within this group. A strong PAX expression is seen in 33.3% (3/9) and moderate expression in 33.3% (3/9). LOH was seen in a higher frequency in 7/9 (79%) of cases.

**WT-1 and p53**

immunohistochemical expression for WT-1 and p53 were similarly seen consistently. A strong nuclear staining is seen in 100% (10/10) of all TIC and 80% (8/10) adjacent invasive carcinoma. LOH was seen in up to 44% (4/9) at the WT-1 locus and 44% (4/9) at the p53 locus.

### 3.3. Group 3: PPSC with Associated TIC (PPSC/+) (n-5)

This group includes all cases of PPSC with associated TIC in the fallopian tubes.

#### 3.3.1. IHC and LOH Results of Group 3

**PAX 2**

there is heterogeneity in the expression of PAX 2 immunostain expression in both TIC as well as PPSC. A moderate-strong staining is seen in 40% (2/5) cases PPSC, and 20% (1/5) of associated TIC. No LOH was seen in both TIC as well as PPSC at the PAX gene.

**WT-1 and p53**

immunohistochemical expression for WT-1 and p53 were similarly seen consistently. A strong nuclear staining is seen in 100% (5/5) of all TIC and 80% (4/5) adjacent invasive carcinoma. No LOH was seen in both TIC as well as PPSC at the WT-1 or p53 gene loci. One (1) out of five (5) cases showed LOH at WT-1 gene locus in both TIC as well as PPSC. Tables 3, 4, and 5 show the immunohistochemical and LOH expression for PAX 2, WT-1, and p53 gene markers.

## 4. Discussion

Primary fallopian tube carcinomas (PFTCs) are rare and comprises up to 1.1% of all gynecologic cancers. In order to be considered for PTFC, the tumor must be macroscopically located within the tube or at the fimbriated end and the uterus and ovary should be free of the tumor. Presence of in situ carcinoma designated as TIC qualifies “fallopian tube” as the origin. A great majority of the tubal carcinomas are predominantly “serous” in histology. Primary peritoneal serous carcinoma (PPSC) is diagnosed in the absence of an “in situ” lesion. The spread of PPSC is similar to ovarian carcinoma and sometimes involvement of adjacent ovary makes it difficult to determine the primary tumor origin and often classified either as ovarian or primary peritoneal in origin based on the “bulk of the tumor.”

Tubal intraepithelial carcinoma (TIC), though fallopian tube in origin has been shown in recent studies to be associated with nearly half of OSC as well as PPSC [3]. Further p53 analysis on both TIC as well as PPSC has shown comparable mutational analysis genetically linking TIC as a precursor lesion to some of the PPSC [7]. These observations were further endorsed in a proposed model for pathogenesis of pelvic serous carcinoma by Crum et al. In this proposed model, when the normal tubal mucosa at the fimbriated end transforms into TIC, the tumor cells exfoliated into the peritoneal surface and thus seeding extensively [8].

Distal fallopian tube (Fimbria) is a unique region with large surface area exposed to biologic events that impact the ovarian surface. It acts as a junction between mesothelium and mullerian epithelium. The reported frequency of PFTC at the fimbriae ranges from 40%–100%. These tumors are diagnosed effortlessly in the presence of a distended fallopian tube with a large centrally located tumor. However most of the tumors in this region are less commonly recognized as PFTC when a microscopic invasive tumor or a focus of TIC is present, until they spread to other sites. Additionally, fallopian tubes are not thoroughly examined in all PPSC for a tubal source. Interestingly, in the tumors arising from the distal fallopian tube though they share a common “precursor lesion” TIC, some of them appear to remain as PFTC and others seed on to the peritoneal surface to become PPSC. One of the primary goals of our study was to understand the biologic properties of the tumors arising in the distal fallopian tube that remain as PFTC are similar when they seed on to the peritoneal surface to become PPSC. Two tumor suppressor gene markers p53 and WT-1 were chosen along with an emerging mullerian marker PAX2 to understand these biologic properties.

*PAX2 *gene, transcription factor expressed in the intermediate mesoderm (*Wolffian ducts as well as müllerian ducts originate*), is located on chromosome 10q24 [9]. PAX2 immunohistochemistry has shown to be a sensitive marker for diagnosing nephrogenic adenomas and renal cell carcinoma [10–12]. In the female genital organs, PAX 2 expression is seen in benign fimbriae of the fallopian tubes to the epithelium of the cervix. In the same study, PAX2 expression is seen in up to (60%) of OSC cases. In a recent published study, we have demonstrated a diffuse PAX 2 expression in simple serous cysts, and serous cystadenofibromas as well as ovarian serous carcinomas [13]. In the same study, we observed a diffuse PAX 2 expression in secretory cells of the tubal epithelial lining [13, 14]. 

P53 is a tumor suppressor gene located on the short arm of chromosome 17. Mutations of p53 are common in many human cancers; overexpression of p53 protein is associated with poor prognosis in a variety of cancers. Mutations of p53 are seen in Type 2, rapidly evolving tumors. P53 mutations are seen in up to 75% (8/12) of p53 signatures and a much higher incidence in TIC as well [7, 12].

*WT-1* gene is consistently detected in both normal ovarian germinal epithelium and human mesothelium. Loss of normal WT-1 gene function is a common event in both PPSC and advanced OSC due to downregulation by regulatory factors. WT-1 immunohistochemical expression was shown in majority of serous carcinomas of ovary, fallopian tube as well as PPSC establishing as a highly sensitive as well as specific marker for tumors of mullerian differentiation [4, 5, 15–17]. In our study, LOH in the WT-1 gene locus was seen in up to 44% of cases in PPSC* without* associated TIC group in contrast to one case (20%) in the PPSC *with *associated TIC.

Using the three markers, we observed a great majority of our PPSC *without *associated TIC exhibited LOH in the PAX gene locus followed by WT-1 gene locus compared to the other tumor groups. In the PFTC group, the LOH patterns of both the TIC as well as the invasive carcinoma were similar. In the tumors of distal fallopian tube with a common TIC origin (that includes PFTC as well as subset of PPSC); there exists a difference in the LOH patterns in the tumors remaining as PFTC in comparison to the tumors that seed on to the peritoneal surface (PPSC). The group of PPSC arising in the absence of TIC exhibits different LOH patterns in comparison to PPSC w/associated TIC. Our study demonstrates the “heterogeneity” that exists within these tumors of the distal fallopian tube. Based on the LOH patterns, it is possible that though these tumors share the same “precursor lesion”, early or late events in genetic stability are facilitating these tumors to follow the pathway of developing into PPSC or to remain as PFTC. It is also possible that the subset of PPSC in the absence of TIC might arise within the mullerian foci of the peritoneal cavity thus circumventing the tubal pathway. We further observed that the TIC in association with PFTC demonstrates different LOH patterns in comparison to PPSC w/TIC also raising the question of tubal origin in some of these tumors.

### 4.1. PAX 2 Signature

Fallopian tube has been shown to be an organ of serous carcinogenesis. Early serous carcinomas were diagnosed in 2%–10% of BRCA (+) women undergoing prophylactic bilateral salpingo-oopherectomies [12, 18]. p53 signatures are defined as benign appearing tubal mucosa that strongly expresses p53 mutations and show low MIB 1 activity. These signatures are present in the distal tube and predominate in “secretory” cells. p53 signature is seen in 24% of BRCA patients and in association with TIC in 50% cases. This signature represents the initiation of serous carcinogenesis in distal fallopian tube and is theorized that women at high genetic risk are more likely to progress from the p53 signature to TIC as well as invasive carcinoma. Though we did not perform MIB 1 in our study to assess the proliferation activity, interestingly in some of our PPSC without TIC group, we have observed a small subset of cases that showed LOH at the PAX 2 locus but no expression by IHC. Though this may not be exactly similar to p53 signature, but appears to be an earliest genetic instability at the PAX 2 locus exhibited by these tumors in comparison to other tumors, might be representative of a type of “PAX 2 signature.” [19] 

## 5. Conclusions

In conclusion, tumors arise in distal FT remaining as PFTC appears to exhibit different LOH patterns when seeds on peritoneal surface (PPSC) though there is no difference in the expression of these tumors by immunohistochemistry. TIC in association with PFTC demonstrates different LOH patterns in comparison to PPSC w/TIC (? Tubal origin). PAX 2 LOH patterns in PPSC represent “PAX signature.” Understanding precursor lesions in distal fallopian tube helps in detection of early serous carcinomas (BRCA). Our study is the first to demonstrate the LOH and IHC of PAX2 in these tumors and might be an emerging biologic marker for early detection. Studies are in progress using these molecular markers in BRCA (+) women w/prophylactic bilateral salpingo-oophorectomies.

## Figures and Tables

**Table 1:** Antibodies, sources, and conditions.

Antibody	Vendor	Reference no.	Clone	Dilution	Pre-Treatment	Detection
WT-1 protein	Cell Marque; Rocklin, CA	CMA788	6F-H2	Pre-dilute	CC1 mild plus Protease 3, 4 min; Vetana Medical	Iview DAB plus Amplification; Ventana
PAX-2	Zymed-Invitrogen; Carlsbad, CA	71–6000	Rabbit Polyclonal	1 : 30	CC1 standard	Iview DAB
P53	Ventana Medical; Tucson, AZ	790–2912	DO-7	Pre-dilute	CC1 standard	Iview DAB

**Table 2:** Clinical and pathologic features of Group 1 [primary fallopian tube carcinoma (PFTC)] arising in the background of tubal intraepithelial carcinoma (TIC).

PFTC (*n*-9)	Age	Histology Diagnosis	Tumor size(cm)	Lymph nodemetastasis		FIGO stage
				Positive	Negative	
Case 1	48	High grade serous	8.0	0	6	T1a
Case 2	81	High grade serous	0.8	0	6	T1a
Case 3	73	High grade serous	5.5	0	9	T2a
Case 4	58	High grade serous	3.0	0	9	T1a
Case 5	79	High grade serous	5.0	0	6	T1a
Case 6	71	High grade serous	4.5	1	4	T3c
Case 7	77	High grade serous	1.6	0	21	T3a
Case 8	44	High grade serous	6.0	6	15	T3c
Case 9	67	High grade serous	1.5	23	0	T3c

PFTC: Primary fallopian tube carcinomas.

**Table 3:** Immunohistochemical (IHC) and loss of heterozygosity (LOH) analysis of Group 1: primary fallopian tube carcinoma (PFTC) and associated tubal intraepithelial carcinoma (TIC).

PPSC/+	Associated TIC						PFTC					
	PAX 2		WT-1		P53		PAX 2		WT-1		P53	
	*IHC (CS)	LOH	*IHC (CS)	LOH	*IHC (CS)	LOH	*IHC (CS)	LOH	*IHC (CS)	LOH	*IHC (CS)	LOH
^♦^Case 1	0	No LOH	7	No LOH	7	**LOH**	0	No LOH	6	No LOH	7	**LOH**
Case 2	0		3		7	No LOH	3	**LOH**	7	No LOH	7	No LOH
Case 3	6		7		0		6	No LOH	7	**LOH**	7	No LOH
Case 4	0		7		7		6	No LOH	7	**LOH**	7	**LOH**
^♦^Case 5	2	**LOH**	7	No LOH	0		3	No LOH	7	No LOH	0	No LOH
^♦^Case 6	7	**LOH**	6	**LOH**	7	No LOH	7	**LOH**	7	No LOH	7	No LOH
Case 7	0		7		7		0	No LOH	7	No LOH	7	No LOH
Case 8	3		7		7		**0	**LOH**	7	No LOH	7	**LOH**
^♦^Case 9	**0	**LOH**	7	**LOH**	**0	**LOH**	0	No LOH	7	**LOH**	0	No LOH
Case 10	6		7		7		6	No LOH	7	No LOH	7	No LOH

*IHC (CS): a cumulative score (CS) was calculated incorporating PS as well as IS. The score was derived by the summation of PS and IS, ranged from 0 to 8 and is further divided into *Negative* (0), *Weak *(1-2), *Moderate *(3–5), *Strong* (6–8).

(**): PAX signature; (^♦^): 4 cases of TIC on which LOH was performed.

**Table 4:** Immunohistochemical (IHC) and loss of heterozygosity (LOH) analysis of Group 2: primary peritoneal serous carcinomas (PPSC) *without *associated tubal intraepithelial carcinoma (TIC).

PPSC/−	PAX 2		WT-1		P53	
	*IHC (CS)	LOH	∗**IHC (CS)**	LOH	*IHC (CS)	LOH
Case 1	7	**LOH**	7	No LOH	7	No LOH
Case 2	**0	**LOH**	7	**LOH**	7	No LOH
Case 3	**0	**LOH**	7	No LOH	7	No LOH
Case 4	7	**LOH**	7	**LOH**	7	**LOH**
Case 5	**0	**LOH**	7	No LOH	7	No LOH
Case 6	7	No LOH	7	**LOH**	3	**LOH**
Case 7	3	**LOH**	7	**LOH**	7	**LOH**
Case 8	3	No LOH	7	No LOH	7	No LOH
Case 9	3	**LOH**	7	No LOH	7	**LOH**

*IHC (CS): a cumulative score (CS) was calculated incorporating PS as well as IS. The score was derived by the summation of PS and IS, ranged from 0 to 8 and is further divided into *Negative* (0), *Weak* (1-2), *Moderate* (3–5), *Strong *(6–8).

(**): PAX signature.

**Table 5:** Immunohistochemical (IHC) and loss of heterozygosity (LOH) analysis of Group 3: primary peritoneal serous carcinomas (PPSC) *with *associated tubal intraepithelial carcinoma (TIC).

PPSC/+	Associated TIC						PPSC					
	PAX 2		WT-1		P53		PAX 2		WT-1		P53	
	*IHC (CS)	LOH	*IHC (CS)	LOH	*IHC (CS)	LOH	*IHC (CS)	LOH	*IHC (CS)	LOH	*IHC (CS)	LOH
Case 1	1	No LOH	7	No LOH	7	No LOH	0	No LOH	7	No LOH	7	No LOH
Case 2	0	No LOH	7	No LOH	7	No LOH	4	No LOH	7	No LOH	7	No LOH
Case 3	5	No LOH	7	No LOH	7	No LOH	6	No LOH	7	No LOH	7	No LOH
Case 4		No LOH	7	No LOH	7	No LOH	0	No LOH	7	No LOH	6	No LOH

*IHC (CS): a cumulative score (CS) was calculated incorporating PS as well as IS. The score was derived by the summation of PS and IS, ranged from 0 to 8 and is further divided into *Negative *(0), *Weak* (1-2), *Moderate* (3–5), *Strong* (6–8).
